# Single Nucleotide Polymorphisms in *Growth Hormone* Gene and Their Association with Growth Traits in *Siniperca chuatsi* (Basilewsky)

**DOI:** 10.3390/ijms15047029

**Published:** 2014-04-22

**Authors:** Changxu Tian, Min Yang, Liyuan Lv, Yongchao Yuan, Xufang Liang, Wenjie Guo, Yi Song, Cheng Zhao

**Affiliations:** Key Lab of Freshwater Animal Breeding, Ministry of Agriculture, Freshwater Aquaculture Collaborative Innovation Center of Hubei Province, College of Fisheries, Huazhong Agricultural University, Wuhan 430070, China; E-Mails: tianchangxu@hotmail.com (C.T.); minyangemma@hotmail.com (M.Y.); lvliyuan2013@gmail.com (L.L.); guowenjie2014@gmail.com (W.G.); songyino222@gmail.com (Y.S.); zhaocheng1307@webmail.hzau.edu.cn (C.Z.)

**Keywords:** *Siniperca chuatsi*, *GH* gene, SNP, growth traits

## Abstract

*Growth hormone* (*GH*) has been considered as a candidate gene for growth traits in fish. In this study, polymorphisms of the *GH* gene were evaluated for associations with growth traits in 282 *Siniperca chuatsi* individuals. Using directly sequencing, four single nucleotide polymorphisms (SNPs) were identified in *GH* gene, with two mutations in *intron 4* (*g.4940A>C*, *g.4948A>T*), one mutation in *exon 5* (*g.5045T>C*) and one in *intron 5* (*g.5234T>G*). Notably, three of them were significantly associated with growth performance, particularly for *g.4940A>C* which was highly correlated with all the four growth traits. In conclusion, our results demonstrated that these SNPs in *GH* gene could influence growth performance of *S.chuatsi* and could be used for marker-assisted selection (MAS) in this species.

## 1. Introduction

The Chinese perch *Siniperca chuatsi* (Basilewsky) is one of the chief economic freshwater fishes for aquaculture in China, with worldwide production of 252,622 tons in 2010 [1]. Due to its delicious flesh, fast-growing and broad temperature tolerance range, nowadays it has been cultured in many provinces of China, mostly in Guangdong Province [2]. However, with the rapid development of the culture industry, some commercially desirable characteristics (growth, disease resistance and suitability) of the cultured stocks have declined [1,3]. These have led commercial breeders to incorporate significant selection for increased growth rate in breeding programs by marker-assisted selection (MAS).

To implement the MAS, markers are usually chosen in genes known to regulate the metabolic network controlling a particular quantitative trait [4]. A statistical association between specific molecular haplotypes (alleles) of a candidate gene and the trait of interest is taken as evidence that the gene is either directly involved in the genetic control of the trait or that the functional polymorphism is sufficiently close to the marker so that the two loci are in linkage disequilibrium (LD) [4]. Generally, most sequence variations are attributable to SNPs, with the rest due to insertion or deletion of one or more bases, repeat length polymorphisms, and rearrangements [5]. With the increase of single nucleotide polymorphisms (SNP) identification speed and efficiency, those studies will promote comprehensive tests of the hypothesis that common variants contribute significantly to the molecular nature of quantitative traits.

Growth traits including growth rate, body weight and length are the most important traits in aquaculture species [6]. *Growth hormone* (*GH*), a major factor in regulating growth of vertebrates, has been considered as a candidate gene associated with linear growth, food conversion and appetite in fish [7–9]. To date, polymorphisms in the *GH* gene have been reported and some SNPs have been revealed to be associated with economic traits in several fishes, such as brown trout [10], chinook salmon [11], bleak [12], Atlantic salmon [13], common bream [14], and large yellow croaker [9].

Although the *GH* gene of *S. chuatsi* has been cloned and sequenced in previous study [15,16], SNP polymorphisms of this gene and its marker-trait association analyses in *S. chuatsi* have not yet been reported. Therefore, the purpose of the current study was to identify and characterize SNPs in *GH* gene and then analyze the association between these polymorphisms and growth traits in the mixed pedigrees of *S. chuatsi* population. These SNPs would provide basic data for MAS in *S. chuatsi*.

## 2. Results and Discussion

### 2.1. Polymorphism Identification and Genotyping

Two hundred and eighty-two individuals were sequenced in this study. In total, four SNPs were identified in *GH* gene, with two mutations in *intron 4* (*g.4940A>C*, *g.4948A>T*), one in *exon 5* (*g.5045T>C*, synonymous mutation) and one in *intron 5* (*g.5234T>G*). The observed heterozygosity (Ho) and expected heterozygosity (He) ranged from 0.3475 to 0.4255 and from 0.4056 to 0.4274, respectively (Table 1). Two loci (*g.4948A>T*, *g.5234T>G*) deviated significantly from Hardy-Weinberg equilibrium (HWE) (*p* < 0.05). Frequencies of three genotypes were presented in Table 2. According to the classification of polymorphism information content (PIC), the mixed pedigrees of *S. chuatsi* population belongs to the median polymorphism level (PIC = 0.33). Based on D’ method in HAPLOVIEW software [17], the estimated values of linkage disequilibrium (LD) analysis between polymorphic fragments within *S. chuatsi* population *GH* gene were calculated (data not shown). The results distributed unevenly in the tested breeds but a LD block between *g.4940A>C* and *g.4948A>T* was consistently observed in the mixed pedigrees.

### 2.2. Association Analysis with Growth Traits

The results of association analysis between different genotypes and growth traits are given in Table 3. At the position of *g.4940A>C*, individuals with the CC genotype had significantly greater values for all analyzed growth traits than those with the AA or AC genotypes (*p* < 0.05). Individuals with the TT genotype at the position of *g.4948A>T* had significantly greater values for body height or total length than those with the AA or AT genotypes (*p* < 0.05). *S. chuatsi* with the CC genotype at the position of *g.5045T>C* had significantly greater values for body length than those with the TC genotype (*p* < 0.05). For the *g.5234T>G*, individuals with the GG genotype had greater values for all analyzed growth traits than those with the TT and TG genotypes, but these differences were not significant (*p* < 0.05). In addition, association analysis is done between different haplotypes and growth traits. However, all of haplotypes have no significant association with any of the measured traits (*p* > 0.05) (data not shown).

### 2.3. Discussion

The candidate gene approach is a very powerful method to investigate associations of gene polymorphisms with economically important traits in aquaculture. The genetic improvement of cultured *S. chuatsi* has been relatively slow compared with other aquaculture fish species [16–18]. So far, few selective breeding programs have been conducted, and no genetic linkage map has been constructed for this species [16]. To our knowledge, there is currently no published literature describing SNP polymorphisms of *GH* gene in fish breeds. The observed DNA variation in the *GH* gene of *S. chuatsi* might directly or indirectly affect growth traits. This potential effect prompted us to investigate the eventual relationships of *GH* gene variation and the growth traits in *S. chuatsi*.

In this study, four novel SNPs were detected in the *GH* gene from 282 *S. chuatsi* individuals. All of the four SNPs presented median polymorphisms, indicating that these SNPs had large genetic variations and selection potentials. The results of the Chi-square test showed that *g.4940A>C* and *g.5045T>C* met the HWE (*p* > 0.05), which indicated that these two loci were under homeostasis accompanied by artificial selection. However, *g.4948A>T* and *g.5234T>G* deviated from the HWE (*p* < 0.05), implying that the selection pressure on these two loci in the population was powerful and effective, or high genetic variation within the population. This may be due to several reasons: (1) the gametes could not combine freely and finally go against HWE; (2) the number of individuals we detected was not sufficient to demonstrate a true event; (3) the growth traits related to *g.5234T>G* were not involved in our study.

In this paper, three SNPs (*g.4940A>C*, *g.4948A>T* and *g.5234T>G*) were tested in introns of *GH* gene and two loci (*g.4940A>C* and *g.4948A>T*) were significantly associated with growth traits in *S. chuatsi.* In the present study, one mutation *g.4940A>C* was striking associated with growth traits of the *S. chuatsi* and that the CC genotype positively affected the four growth traits. The SNP *g.4948A>T* was found to be significantly associated with growth traits, with the TT genotype positively affecting body height. Furthermore, the SNP *g.5234T>G* was a polymorphism likely associated with growth traits, with the GG genotype having positive effects on growth traits, even not significant. Hence, SNPs in *GH* introns are likely to play important roles in the gene’s transcription, translation, and expression and could thus influence the growth and developmental stages of the fish. In the study, a LD block between *g.4940A>C* and *g.4948A>T* was observed in the mixed pedigrees, indicating that mutations in introns of *GH* gene may be LD with one or more nearby quantitative trait locus (QTL) associated with growth traits. The SNP *g.5045T>C* was found to be significantly associated with body length, with the CC genotype positively affecting such growth trait. This locus was synonymous mutation in *exon 5*, and two hypotheses could explain how synonymous mutations may affect growth traits: (1) synonymous mutations may indirectly affect gene functions by affecting alternative splicing, splicing efficiency, messenger RNA turnover, and subsequent gene expression; or (2) synonymous mutations may be LD with one or more nearby QTL associated with growth traits [19,20].

A haplotype is a physical arrangement of SNP alleles along a chromosome [21]. With the availability of high density SNP markers, haplotypes play an important role in association studies and also provide insight on factors influencing the dependency among SNPs [22]. However, all of haplotypes have no significant association with any of the measured traits in the study, indicating that the statistically association may not mean the *GH* gene has a direct involvement in growth traits, as the quantitative loci may be located somewhere on the same chromosome arm as the *GH* gene.

This research may serve as the first step towards the implementation of MAS strategies to improve the productivity and have potential applications in future genetic improvement of growth performance in *S. chuatsi*. However, developing the high performing culture lines is a complex program, and further validation will be necessary to confirm many pivotal details before the SNPs can contribute to the MAS program for genetic improvement of *S. chuatsi.* To validate the associations, further investigation should be repeated in larger independent populations from various environments, and several traits such as yield, meat quality and disease resistance should be used during the selection objective.

## 3. Experimental Section

### 3.1. Samples Collection and Preparation

Total 282 individuals (approximately age 1 year) in the mixed pedigrees of *S. chuatsi* population were randomly obtained from the Qingyuan fish farm in Guangdong province of China in October 2012. All experimental fish were maintained under constant environmental conditions and were fed according to the management practices of the fishery farm. Before the experiment, four growth traits (body weight, total length, body length and body height) were recorded for statistical analysis. Tissues were collected from fin clips and preserved in 95% ethanol at −20 °C until processing for DNA isolation. Total genomic DNA was extracted from fin clips using the TIANamp Genomic DNA Kit (Tiangen, Beijing, China) following the manufacturer’s instructions.

### 3.2. Primer Design and Polymerase Chain Reaction (PCR) Amplification

Three pairs of primers were designed using Primer Premier 5 software (PRIMER Biosoft International, Palo Alto, CA, USA) to amplify the *S. chuatsi GH* gene partial regions (Table 3).

Polymerase chain reaction (PCR) amplifications were performed in 50 μL reaction volumes containing 5 μL of 10× PCR buffer (Mg^2+^ Plus), 5 μL dNTPs (2.5 mM each), 2 μL of each primer (10 μM), 0.5 μL of Taq DNA polymerase (5 U/μL, TaKaRa, Dalian, China) and 1 μL of genomic DNA (50–100 ng/μL). PCR conditions were as follows: initial denaturation at 94 °C for 3 min followed by 30 cycles at 94 °C for 30 s, the optimized annealing temperature (Table 1) for 45 s, 72 °C for 30 s, and then a final extension step at 72 °C for 10 min. Amplification results were verified by 1.5% agarose gel electrophoresis. PCR fragments of the predicted size were cut and purified from gels with an agarose gel DNA Extraction kit (TaKaRa, Dalian, China).

### 3.3. Single Nucleotide Polymorphism Identification and Genotyping

Fragments in 282 individuals were sequenced by direct sequencing using an ABI 3730 DNA Analyzer (Applied Biosystems, Foster City, CA, USA). Sequence mutations differing between individuals were detected using the ClustalX Multiple Sequence Alignment Program (version 1.83) [23]. SNPs were identified and genotyped with DNAStar software (version 7.1, DNASTAR, Madison, WI, USA).

### 3.4. Statistical Analysis

The genotype frequencies were calculated and HWE was tested using a chi-square test of PopGene32 [24]. The population genetic indexes including He, Ho, effective allele numbers (Ne) and PIC were calculated by Nei’s method [25]. Generally, polymorphism information content (PIC) is classified in to the following three types: low polymorphism (PIC value < 0.25), median polymorphism (0.25 < PIC value < 0.5) and high polymorphism (PIC value > 0.5). The LD structure measured by D’ and *r*^2^ was performed with the HAPLOVIEW software (Ver.3.32) [26]. Association analyses between genotypes or haplotypes of *GH* gene and four growth traits were performed using general linear model (GLM) procedure with SPSS 17.0 software (IBM, Armonk, NY, USA). We used the following statistical model:

(1) Y=u+G+e

where *Y* is the phenotypic value of each trait; *u* is population mean value of 4 growth traits, *G* is the fixed genotype effect of each SNP, and *e* is the random error effect. Multiple comparisons between different genotypes were tested using the LSD method with Bonferroni correction adjustment [27].

## 4. Conclusions

In conclusion, four novel SNPs were found in *S. chuatsi GH* gene. And three of them were significantly associated with growth performance. This may suggest that these SNPs could affect growth performance in *S. chuatsi*, and can be employed in MAS for the purpose of advancing *S. chuatsi* breeding and genetic research.

## Figures and Tables

**Table 1.** Diversity parameters of four single nucleotide polymorphisms (SNPs) within the *S. chuatsi Growth hormone* (*GH*) gene.

Locus	Ne	He	Ho	PIC	*p*-value (HWE)
*g.4940A>C*	1.74	0.4261	0.3865	0.3349	0.525
*g. 4948A>T*	1.68	0.4247	0.3475	0.3341	0.017*
*g. 5045T>C*	1.74	0.4056	0.3723	0.3229	0.212
*g.5234T>G*	1.74	0.4274	0.4255	0.3356	0.008*
Mean	1.72	0.4209	0.3830	0.3319	0.191

Ne, effective allele numbers; He, expected heterozygosity; Ho, observed heterozygosity; PIC, polymorphism information content;

* significant at the *p* < 0.05 level;

HWE, Hardy-Weinberg equilibrium.

**Table 2.** Association between four polymorphic SNP sites of *GH* gene and growth traits in *S. chuatsi*.

Locus	Genotype	Genotypic frequencies	Body weight (g)	Total length (cm)	Body length (cm)	Body height (cm)
*g.4940A>C*	AC (109)	0.39	477.70 ± 39.95^a^	31.40 ± 0.83^a^	27.17 ± 0.68^a^	9.69 ± 0.30^a^
	AA (142)	0.50	482.52 ± 20.02^a^	31.65 ± 0.54^a^	27.64 ± 0.44^a^	9.89 ± 0.20^a^
	CC (32)	0.11	584.89 ± 41.02^b^	35.25 ± 0.85^b^	30.80 ± 0.70^b^	10.95 ± 0.31^b^

*g. 4948A>T*	AA (147)	0.52	480.66 ± 20.86	31.56 ± 0.57^a^	27.60 ± 0.47	9.85 ± 0.20^a^
	AT (98)	0.35	494.84 ± 37.90	32.03 ± 0.81a,b	27.70 ± 0.66	9.86 ± 0.28^a^
	TT (37)	0.13	531.64 ± 39.76	33.34 ± 0.84^b^	28.95 ± 0.69	10.48 ± 0.30^b^

*g. 5045T>C*	TC (105)	0.37	484.27 ± 43.20	31.61 ± 0.91	27.34 ± 0.75^a^	9.77 ± 0.32
	TT (150)	0.53	495.64 ± 26.29	32.01 ± 0.55	27.94 ± 0.45^b^	9.99 ± 0.19
	CC (27)	0.10	504.07 ± 44.59	33.06 ± 0.94	29.00 ± 0.78^b^	10.30 ± 0.34

*g.5234T>G*	TT (135)	0.48	487.32 ± 25.92	31.74 ± 0.92	27.68 ± 0.45	9.90 ± 0.19
	TG (120)	0.43	492.96 ± 43.55	31.97 ± 0.55	27.76 ± 0.76	9.90 ± 0.33
	GG (27)	0.10	514.01 ± 44.01	33.07 ± 0.71	28.74 ± 0.77	10.23 ± 0.34

a,b , Values with different superscripts within the same column differ significantly at *p* < 0.05; Same superscript or no marked means no difference.

**Table 3.** Primer sets used for the identification of single nucleotide polymorphisms (SNPs) in the *growth hormone* (*GH*) gene in *S. chuatsi*.

Primer name	The size of the fragment (bp)	Primer sequence (5′–3′)	Location along the gene	Amplified gene frament	Annealing temperature (°C)
G1	465	F: GCAACCCGATGAGAAATA	163–627	Part of *intron 2*, *exon 2*, part of *intron 3*	55
		R: CTCTGCGAGCTGCTGTAA			
G2	919	F: GGAAAGGCAGAATGGATG	3111–4029	Part of *intron 3*, *exon 3*, part of *intron 4*	55
		R: GAGGCTCAGATGATTGTTGGTC			
G3	516	F: GAGTTTCCCAGTCGTTCT	4827–5342	Part of *exon 4*, *intron 4*, *exon 5*, *intron 6*, *exon 6*, part of *3′-UTR*	54
		R: GCGTGGCTTCACAGTAG			
